# Preventive Effects of Safety Helmets on Traumatic Brain Injury after Work-Related Falls

**DOI:** 10.3390/ijerph13111063

**Published:** 2016-10-29

**Authors:** Sang Chul Kim, Young Sun Ro, Sang Do Shin, Joo Yeong Kim

**Affiliations:** 1 Department of Emergency Medicine, Konkuk University School of Medicine and Chungju Hospital, Chungju 27376, Korea; arahan@kku.ac.kr; 2 Laboratory of Emergency Medical Services, Seoul National University Hospital Biomedical Research Institute, Seoul 03080, Korea; shinsangdo@gmail.com (S.D.S.); blj01he@gmail.com (J.Y.K.); 3 Department of Emergency Medicine, Seoul National University College of Medicine, Seoul 03080, Korea; 4 Department of Emergency Medicine, Korea University Ansan Hospital, Ansan 15355, Korea

**Keywords:** occupational injuries, accidental falls, traumatic brain injury

## Abstract

Introduction: Work-related traumatic brain injury (TBI) caused by falls is a catastrophic event that leads to disabilities and high socio-medical costs. This study aimed to measure the magnitude of the preventive effect of safety helmets on clinical outcomes and to compare the effect across different heights of fall. Methods: We collected a nationwide, prospective database of work-related injury patients who visited the 10 emergency departments between July 2010 and October 2012. All of the adult patients who experienced work-related fall injuries were eligible, excluding cases with unknown safety helmet use and height of fall. Primary and secondary endpoints were intracranial injury and in-hospital mortality. We calculated adjusted odds ratios (AORs) of safety helmet use and height of fall for study outcomes, and adjusted for any potential confounders. Results: A total of 1298 patients who suffered from work-related fall injuries were enrolled. The industrial or construction area was the most common place of fall injury occurrence, and 45.0% were wearing safety helmets at the time of fall injuries. The safety helmet group was less likely to have intracranial injury comparing with the no safety helmet group (the adjusted odds ratios (ORs) (95% confidence interval (CI)): 0.42 (0.24–0.73)), however, there was no statistical difference of in-hospital mortality between two groups (the adjusted ORs (95% CI): 0.83 (0.34–2.03). In the interaction analysis, preventive effects of safety helmet on intracranial injury were significant within 4 m height of fall. Conclusions: A safety helmet is associated with prevention of intracranial injury resulting from work-related fall and the effect is preserved within 4 m height of fall. Therefore, wearing a safety helmet can be an intervention for protecting fall-related intracranial injury in the workplace.

## 1. Introduction

Work-related injuries are public health issues on a global scale, and they lead to exorbitant medical and social costs as well as to a loss of healthy life and productivity [1,2]. Work-related fall was the second leading cause of death among work-related injuries after motor vehicle crashes [3]. By the mechanism of injury, the fatal work-related fall injuries accounted for 20% in Korea and 14% in the US among all case-fatality after work-related injuries [4,5]. The economic cost from nonfatal work-related fall injuries in the US was nearly 16 billion USD per year, and over 25% of fall injuries resulted in 31 or more workdays being lost [6,7,8].

Traumatic brain injury (TBI) is a common cause of case-fatality, cognitive impairment, and post-injury functional disability [9,10]. Furthermore, even mild TBI can have long-term consequences [11]. Severe TBI is a catastrophic event that can potentially result in a devastating socioeconomic life since the sequelae affects multiple aspects of daily life; however, there was no evidence showing that therapeutic interventions after suffering severe TBI can effectively improve the functional outcome [12,13,14]. Therefore, efforts directed towards awareness of hazard and injury prevention are emphasized to reduce the public health burden of TBI. Work-related TBI is caused mainly by falls, motor vehicle crashes, and assaults in manufacturing and construction industries, and it is avoidable by developing preventive measures. However, interventions with the goal of preventing TBI resulting from fall injuries are not available in the current workplace environment.

Safety helmets are useful protective equipment, which reduce the risk of TBI and death resulting from sports activities as well as motorcycle and bicycle accidents [15,16,17]. However, the preventive effect of safety helmets on health outcomes resulting from work-related fall injuries has not been verified mainly because safety helmets have been primarily used to prevent workers from experiencing head injuries caused by falling or flying objects. This has been the case in several countries, such as the US, UK, EU, Japan, and Australia [18].

Safety precautions that focus on reducing the risk of TBI resulting from fall injuries are limited in the current workplace environment. In addition, research studies that focus on the effect of safety helmets on reducing the risk of TBI resulting from work-related fall injuries are rare. We hypothesize that safety helmets will have a preventive/positive effect on reducing the risk of TBI resulting from a fall within a certain height. Hence, this study aims to evaluate the extent to which safety helmets have an effect on reducing the risk of TBI resulting from work-related fall injuries by comparing the effect across different heights.

## 2. Methods

### 2.1. Study Design and Setting

This is an observational study that uses the Emergency Department-based Occupational Injury Surveillance (EDOIS) database in Korea. The EDOIS is a nationwide, prospective database of work-related injury patients who visit the emergency department (ED). The ED gathers injury-related and workplace information, which are used to plan and develop national policies concerning work-related injury prevention. The EDOIS project was organized and financially supported by the Korea Occupational Safety and Health Agency (OSHA), and a total of 10 emergency departments participated in this project. The emergency departments were chosen by using a stratified sampling method based on the ED’s geographical location and level.

### 2.2. Data Source and Collection

The EDOIS was designed to include all work-related (only paid work) injuries. The database collects the patients’ demographic information, occupation and workplace information, injury-related information, prehospital information, and ED and hospital information. Primary surveillance and data collection were performed by general physicians after obtaining informed consent, and most of the recorded information was supervised and modified on a daily basis by emergency physicians and trained research coordinators. An occupational medicine doctor in each participating hospital reviewed all of the surveillance database on a monthly basis to confirm whether the injury was associated with paid work and to give further advice regarding treatment for the work-related injuries.

The project’s quality management committee established survey guidelines, which included definitions of occupational terms, definitions of survey protocols, definitions of codes, and classification of data variables. Furthermore, the committee also trained all research coordinators in each study hospital prior to joining this project. All research coordinators had to input surveillance data using Microsoft Access, and the quality management committee reviewed the data on a monthly basis and provided regular feedback in order to maintain data quality.

### 2.3. Study Population

The study population included all the adult patients who experienced work-related fall injuries and visited any ED of the 10 study hospitals (five level 1 EDs and five level 2 EDs) between July 2010 and October 2012. We excluded cases in which the information regarding the height of fall, the presence of safety devices at the time of the fall, or the resulting clinical outcomes was unknown. Patients who visited EDs due to recurring post-injury complications were not included in this study.

Work-related fall injuries consist of fall to lower level, which is further broken down into subcategories including fall from step or ladder, fall from existing floor openings, fall from a stack of luggage or payload, fall from roof, fall from scaffolding, fall from building girders or other structural steel, fall from non-moving vehicle or machine equipment, and etc.

### 2.4. Main Outcomes

The primary endpoint was intracranial injury, defined according to the diagnosis of International Classification of Diseases, Tenth Revision (ICD-10) code S06.1–S06.9 which is recorded on the discharge summary after ED and/or hospital admission. The secondary endpoint was in-hospital mortality, defined as death in ED or during initial admission resulting from the injury regardless of the duration from injury to death, and it is determined at the point of discharge from ED or hospital.

### 2.5. Variables and Measurements

The main exposure variable was the use of safety helmets, which is detected by the EDOIS registry.

We collected information on the demographic factors (age, gender, education level, and annual income for the past year), occupation and workplace information (occupation, individual career related to work, type of employment, working type, personal protective equipment (PPE) including safety devices and facilities, safety education enrollment status, and construction-related work), injury-related information (time of injury, place of injury, height of fall, and cause of the incident), and prehospital, ED, and hospital information (mode of transportation, prehospital treatment, clinical findings, diagnostic assessment and medical treatments in the ED, ED disposition, patient outcome after admission if the patient was admitted, expected days away from work, and convalescence via the workers’ compensation insurance).

An occupation was categorized into 10 major groups based on the international standard classification of occupations (ISCO-08). The occupation categories were the following: (1) Managers; (2) Professionals; (3) Technicians and associate professionals; (4) Clerical support workers; (5) Service and sales workers; (6) Skilled agricultural, forestry and fishery workers; (7) Craft and related trades workers; (8) Plant and machine operators, and assemblers; (9) Elementary occupations; and (10) Armed forces occupations. Types of employment were divided into contracted workers (permanent or temporary) and daily worker. The working type was divided into lone work and cooperated work. Lone work is a type of work in which an employee undertakes an unaccompanied work activity and/or without direct or close supervision [19]. On the other hand, cooperative work is defined as a type of work in which an employee works with a colleague nearby. Place of injury was further categorized into industrial or construction area, farm or other place of primary production, transport area (road), home and residential institution, and other public areas including school, sports and recreational area, and public building, based on the International Classification of External Causes of Injuries (ICECI version 1.2, World Health Organization, Geneva, Switzerland).

“Expected days away from work” indicates the expected time duration for loss of paid work caused by work-related injuries. This is based on the medical certificate, which is measured by an emergency medicine physician. Convalescence by workers’ compensation insurance is the duration required for recovery from work-related injury based on diagnosis and operation by the Industrial Accident Compensation Insurance Act.

### 2.6. Statistical Analysis

The study population was divided into two groups. One group had safety helmets and the other had no safety helmets. Categorical variables were expressed as counts and proportion; continuous variables were expressed as the median and inter-quartile range (IQR). Differences between the two groups were compared using the Pearson’s chi-square test and the Mann–Whitney test.

Adjusted odds ratios (ORs) with 95% confidence intervals (95% CIs) of helmet use for the study endpoints were calculated using multivariable logistic regression analysis with no helmet use as reference. The model adjusted for age, gender, education level, annual income, occupation, safety education enrollment status, type of employment, working type, time of injury, and place of injury.

To determine variability of the preventive effect of safety helmets on study endpoints according to different heights of work-related fall, we developed an interaction model using an interaction term (safety helmet × height of fall) as the final multivariable logistic regression model. The criterion for the *p*-value was defined as a two-sided significance level of 0.05. All statistical analysis was performed using SAS software, version 9.4 (SAS Institute Inc., Cary, NC, USA).

### 2.7. Ethics Statements

The study was reviewed and approved by the Institutional Review Board of Seoul National University Hospital (IRB No. 1204-009-403). Written informed consent was obtained from all participants before initiating the study.

## 3. Results

Among 1651 patients who suffered from work-related fall injuries, 1298 (78.6%) patients were enrolled for this study. Cases who had unavailable information regarding the height of fall (*n* = 305, 22.7%) and safety helmet (*n* = 48, 2.9%) were excluded.

Table 1 shows the demographic characteristics by safety helmet use. Among 1298 eligible patients, 584 (45.0%) were wearing safety helmets at the time of fall injuries. The proportion of female, contracted worker, lone work, and no safety education at workplace were less likely to wear safety helmets (all *p* < 0.0001). The industrial or construction area was the most common place of fall injury occurrence, and falls from step or ladder, building girders or other structural steel, and scaffolding or staging were common in detailed fall mechanisms. The median height of fall was 2.2 meters, and height for the safety helmet group was higher than that of the no safety helmet group (median 3 m vs. 2 m, *p* < 0.0001). In terms of clinical outcomes, the no safety helmet group had a higher proportion of intracranial injury (8.7% vs. 4.6%, *p* = 0.004); however, there was no statistical difference in terms of in-hospital mortality (2.9% vs. 3.1%, *p* = 0.882). Expected days away from work were longer in the safety helmet group (28 days vs. 20 days, *p* = 0.0005); in contrast, convalescence by worker’s compensation insurance was longer in the no safety helmet group (six weeks vs. five weeks *p* < 0.0001) (Table 1).

Table 2 shows the demographic characteristics by intracranial injury. The proportion of wearing safety helmets was higher in no intracranial injury patients (46.1%) than in intracranial injury patients (30.3%) (*p* = 0.004). The medians heights of fall were 2.5 m for intracranial injury patients and 2 m for no intracranial injury patients (*p* = 0.332) (Table 2).

The results for the multivariable logistic regression models, including adjusted ORs (95% CIs) for safety helmet and height of fall, are shown in Table 3. The safety helmet group was less likely to have intracranial injury after work-related falls comparing with the no safety helmet group (the adjusted ORs (95% CI): 0.42 (0.24–0.73)). There was no statistical difference in terms of in-hospital mortality (the adjusted ORs (95% CI): 0.83 (0.34–2.03)). In contrast, the height of fall was associated with increased risk for in-hospital mortality; however, there was no statistical difference in intracranial injury (Table 3).

In the interaction model in which the preventive effects of safety helmets were determined according to different heights of fall, while there were significant preventive effects of safety helmet on intracranial injury when the height of fall was less than 4 m (the adjusted ORs (95% CI): 0.21 (0.05–0.96) from less than 2 m and 0.37 (0.18–0.77) from the height of 2 m to 4 m), there was no statistical difference between the safety helmet group and the no safety helmet group who suffered fall injury from a height of more than 4 m. There was no statistical difference regarding in-hospital mortality between the safety helmet group and the no safety helmet group in any height of fall (Table 4).

## 4. Discussion

This study identified significant preventive effects of safety helmets on intracranial injuries resulting from work-related falls; however, they had no significant effects to reduce in-hospital mortalities. Only 45% of work-related fall injured patients were wearing safety helmets, and the intracranial injury was high at 8.7% in the no safety helmet group and 4.6% in the safety helmet group, respectively. According to heights of fall, the preventive effects of the safety helmets on intracranial injury resulting from work-related falls were preserved within a height of four meters. Wearing a safety helmet in the workplace could reduce intracranial injuries resulting from work-related fall injuries.

The common primary functions of safety helmets in workplace are shock absorption from falling or flying subjects, protection against electrical shock and from flame, and resistance to various working temperature [18,20]. Because the preventive effect of safety helmets on clinical outcomes resulting from work-related fall injuries has not been verified, the function of head protection against fall injury is excluded in most countries’ workplace regulations including Japan, Europe, Australia, and the US [18]. In Korea, in contrast, AB or ABE type of helmet (including B symbols) among A, AB, AE, and ABE are manufactured for protection against TBI resulting from fall injuries as well as caused by falling or flying objects without obvious evidences. In this study, the safety helmet group were less likely to have intracranial injuries after work-related falls, and this results could be one of the evidences to regulate the wearing of safety helmet in workplaces for purposes of preventing of intracranial injuries resulting from work-related fall injuries.

Helmet use would lessen the impact from primary collision and prevent second collision of human body to other structures. It has been verified that helmets have a significant effect to reduce the risk of TBI and case-fatality for patients injured from motorcycle and bicycle crash [16,17]. However, safety helmet had no significant effects on in-hospital mortalities in this study. There was more severe injury in patients wearing safety helmet. (Table 1) Fatality of fall injury may depend on height of fall, the hardness of floor surface and the first body part to contact with the floor [21,22,23].

The height of fall is one of the main influencing factors of clinical outcomes after work-related falls. As heights of fall increase, the injury severity and case-fatality also increased by the effect of height energy on the stained body. In this study, the height from which the fall occurred was significantly higher in those with safety helmets. People were more likely to wear a helmet when their perception of danger was greater. However, the preventive effects of the safety helmets on intracranial injury resulting from work-related falls were retained for only within a height of 4 m. About 28% of case-fatality patients fall from a ladder occurred at a height of 1.8–3 meters [24,25]; therefore, wearing safety helmets should be recommended for all workers working at low height.

Case-fatalities from falls in construction occupies half of all fatal falls in all industries according to US Bureau of Labor Statistics [26]. In this study, industrial or construction area (62.9%) was the most common place of fall injuries, followed by public place (12.8%) and farm or other place of primary production (10.7%). Among all cause of falls, falls from step or ladder was the most common site of work-related fall in this study (Table 1). Other studies reported that falls from building girders or other structural steel, scaffolding, staging, ladder, and existing floor openings are common cause of fall sites for construction workers [27]. Therefore, a worker working at step or ladder in industrial or construction area should wear a proper safety helmet for protection against intracranial injury.

Despite a decline in the incidence rate of overall work-related injury, the Korean case-fatality rate was still 5.8 per 100,000 workers in 2014 which was higher than 3.4 per 100,000 full-time equivalent workers in 2014 in the US [4,5]. A work-related fall was one of the fatal mechanisms of work-related injuries. Intervention strategies, including enforcement of regulations, surveillance system, changes in PPE and safety facilities, and mandatory structured safety education and training, should be developed and evaluated to reduce public burden from work-related fall injuries [28]. In terms of a facility and PPE, active measures including surface protections (non-slip flooring), fixed barriers, and surface opening protections (hole coverings) as well as passive measures including travel restraint systems (safety belt), fall arrest systems (safety harness), and fall containment systems (safety nets) are recommended to decrease or inhibit injury after an initiated fall [29]. In addition, direct interventions, including workplace campaign and awareness for importance of wearing safety helmets and development of new design of safety helmets having protective effect of TBI at over 4 m height of fall, is needed to prevent intracranial injury resulting from work-related fall injuries.

### Limitation

This study has several limitations. Firstly, this is an observational study; there may been a potential confounder that exerted an impact. Well-designed propensity scoring analysis and a systematic review with meta-analysis would be useful to control it and model causal inference. Secondly, the safety helmet, which was the main exposure variable, was measured by face-to-face interview by general physicians. Furthermore, we did not collect information on which type of safety helmet the subjects wore on at the fall incident, or information on whether the helmet was worn appropriately. Occupational safety helmet does not always provide sufficient protection against falls and other events that may lead to a TBI as helmets are often strapless and may come off during a fall. Wearing a helmet with a chinstrap anchored to three points (two sides and rear positioned the other one) can keep the helmet on the right position of worker’s head even in a tumbling fall [30]. Thirdly, we analyzed the preventive effect on the intracranial head injury of safety helmet not by the impact of measured fall energy, but by patient’s stating fall height. We did not consider the energy decrease by contacting another structure during fall.

## 5. Conclusions

A safety helmet is associated with prevention of intracranial injury resulting from work-related fall and the effect is preserved within 4 m height of fall. Development of safer helmet and regulation for wearing safety helmet firmly might be interventions for protecting against fall-related TBI in the workplace. Effective prevention strategy including providing enough fall protection PPE (safety belt, harness, and lifeline) to prevent a worker from falling and safety training/education about fall protection for worker is necessary for both of employers and employees in workplace.

## Figures and Tables

**Table 1:** Demographic findings of study population by safety helmet groups.

Characteristics	Total		Safety Helmet		No Safety Helmet		*p*-Value
	*N*	%	*n*	%	*n*	%	
Total	1298	100.0	584		714		
Patient characteristics							
Age, year							0.578
18–29	77	5.9	35	6.0	42	5.9	
30–49	589	45.4	272	46.6	317	44.4	
50–64	548	42.2	245	42.0	303	42.4	
65 and over	84	6.5	32	5.5	52	7.3	
Median (IQR)	49 (41–56)		49 (41–55)		49 (41–56)		0.055
Gender							<0.0001
Male	1207	93.0	569	97.4	638	89.4	
Female	91	7.0	15	2.6	76	10.6	
Occupation							<0.0001
Managers	14	1.1	7	1.2	7	1.0	
Professionals	23	1.8	6	1.0	17	2.4	
Technicians and associate professionals	174	13.4	106	18.2	68	9.5	
Clerical support workers	22	1.7	4	0.7	18	2.5	
Service and sales workers	74	5.7	8	1.4	66	9.2	
Skilled agricultural, forestry and fishery workers	84	6.5	7	1.2	77	10.8	
Craft and related trades workers	378	29.1	211	36.1	167	23.4	
Plant and machine operators and assemblers	107	8.2	30	5.1	77	10.8	
Elementary occupations	387	29.8	195	33.4	192	26.9	
Other	35	2.7	10	1.7	25	3.5	
Educational level							0.001
Middle school or below	389	30.0	177	30.3	212	29.7	
High school	596	45.9	296	50.7	300	42.0	
College or above	179	13.8	70	12.0	109	15.3	
Unknown	134	10.3	41	7.0	93	13.0	
Annual income, USD							0.001
0–30,000	631	48.6	291	49.8	340	47.6	
Over 30,000	324	25.0	169	28.9	155	21.7	
Unknown	343	26.4	124	21.2	219	30.7	
Occupation and workplace information							
Type of employment							<0.0001
Contracted worker	652	50.2	266	45.5	386	54.1	
Daily worker	573	44.1	296	50.7	277	38.8	
Other	73	5.6	22	3.8	51	7.1	
Working type							<0.0001
Lone work	402	31.0	118	20.2	284	39.8	
Cooperative work	896	69.0	466	79.8	430	60.2	
Safety education enrollment							<0.0001
Yes	680	52.4	429	73.5	251	35.2	
No	543	41.8	138	23.6	405	56.7	
Unknown	75	5.8	17	2.9	58	8.1	
Construction-related work							<0.0001
Yes	383	29.5	254	43.5	129	18.1	
Injury characteristics							
Height of fall injury, meter							<0.0001
0–2	370	28.5	116	19.9	254	35.6	
2–4	595	45.8	275	47.1	320	44.8	
4–6	192	14.8	100	17.1	92	12.9	
Over 6	141	10.9	93	8.2	48	6.7	
Median (IQR)	2.2 (1.5–4.0)		3.0 (2.0–4.0)		2.0 (1.5–3.0)		<0.0001
Time of injury							<0.0001
Scheduled job	1200	92.4	550	94.2	650	91.0	
Extended/holiday work	70	5.4	33	5.7	37	5.2	
Other	28	2.2	1	0.2	27	3.8	
Place of injury							<0.0001
Industrial or construction area	816	62.9	450	77.1	366	51.3	
Farm or other place of primary production	139	10.7	46	7.9	93	13.0	
Transport area (road)	70	5.4	20	3.4	50	7.0	
Other public area	166	12.8	26	4.5	140	19.6	
Home and residential institution	107	8.2	42	7.2	65	9.1	
Cause of the incident							<0.0001
Fall from step or ladder	393	30.3	166	28.4	227	31.8	
Fall from existing floor openings	67	5.2	32	5.5	35	4.9	
Fall from a stack of luggage or payload	93	7.2	37	6.3	56	7.8	
Fall from roof	78	6.0	26	4.5	52	7.3	
Fall from scaffolding or staging	174	13.4	111	19.0	63	8.8	
Fall from building girders or other structural steel	210	16.2	142	24.3	68	9.5	
Fall from nonmoving vehicle or machine equipment	208	16.0	61	10.4	147	20.6	
Other	75	5.8	9	1.5	66	9.2	
Clinical outcomes							
Mode of transportation							0.048
EMS use	888	68.4	416	71.2	472	66.1	
Head injury							
Head injury (S06.0–S06.9)	174	13.4	67	11.5	107	15.0	0.065
Intracranial injury (S06.1–S06.9)	89	6.9	27	4.6	62	8.7	0.004
Anatomical classification of injury							
Head, face, neck	597	46.0	236	40.4	361	50.6	<0.0001
Thorax	285	22.0	132	22.6	153	21.4	0.611
Abdomen	325	25.0	164	28.1	161	22.5	0.022
Extremity	640	49.3	287	49.1	353	49.4	0.916
External and other	112	8.6	78	13.4	34	4.8	<0.0001
Multiple injury							0.038
More than 2 anatomical region	513	39.5	249	42.6	264	37.0	
Operation							0.047
Yes	343	26.4	170	29.1	173	24.2	
ED disposition							0.008
Discharge	409	31.5	159	27.2	250	35.0	
Transfer to other hospital	131	10.1	70	12.0	61	8.5	
Admission	739	56.9	344	58.9	395	55.3	
Death	19	1.5	11	1.9	8	1.1	
In-hospital mortality							
Total	39	3.0	18	3.1	21	2.9	0.882
In ED	19	1.5	11	1.9	8	1.1	
In Ward or ICU	20	2.7	7	2.0	13	3.3	
Expected days away from work							0.001
Median (IQR)	21 (7–42)		28 (8–46)		20 (5–40)		
Convalescence by insurance, week							<0.0001
Median (IQR)	6 (2–8)		5 (2–8)		6 (3–10)		

IQR: interquartile range; EMS: emergency medical services; ED: emergency department; ICU: intensive care unit.

**Table 2:** Demographic findings of study population by intracranial injury groups.

Characteristics	Total		Intracranial Injury		No Intracranial Injury		*p*-Value
	*N*	%	*n*	%	*n*	%	
Total	1298	100.0	89		1209		
Helmet							0.004
Safety helmet	584	45.0	27	30.3	557	46.1	
No safety helmet	714	55.0	62	69.7	652	53.9	
Height of fall injury, meter							0.328
0–2	370	28.5	18	20.2	352	29.1	
2–4	595	45.8	44	49.4	551	45.6	
4–6	192	14.8	15	16.9	177	14.6	
Over 6	141	10.9	12	13.5	129	10.7	
Median (IQR)	2.2 (1.5–4.0)		2.5 (2.0–4.0)		2.0 (1.5–4.0)		0.332
Age, year							0.598
Median (IQR)	49 (41–56)		50 (41–57)		49 (41–56)		
Gender							0.744
Male	1207	93.0	82	92.1	1125	93.1	
Type of employment							0.630
Contracted worker	652	50.2	43	48.3	609	50.4	
Daily worker	573	44.1	39	43.8	534	44.2	
Other	73	5.6	7	7.9	66	5.5	
Safety education enrollment							0.405
Yes	680	52.4	45	50.6	635	52.5	
No	543	41.8	36	40.4	507	41.9	
Unknown	75	5.8	8	9.0	67	5.5	
Construction-related work							0.305
Yes	383	29.5	22	24.7	361	29.9	
Place of injury							0.986
Industrial or construction area	816	62.9	57	64.0	759	62.8	
Farm or other place of primary production	139	10.7	9	10.1	130	10.8	
Transport area (road)	70	5.4	5	5.6	65	5.4	
Other public area	166	12.8	12	13.5	154	12.7	
Home and residential institution	107	8.2	6	6.7	101	8.4	
Cause of the incident							<0.0001
Fall from step or ladder	393	30.3	29	32.6	364	30.1	
Fall from existing floor openings	67	5.2	3	3.4	64	5.3	
Fall from a stack of luggage or payload	93	7.2	10	11.2	83	6.9	
Fall from roof	78	6.0	5	5.6	73	6.0	
Fall from scaffolding or staging	174	13.4	8	9.0	166	13.7	
Fall from building girders or other structural steel	210	16.2	16	18.0	194	16.0	
Fall from nonmoving vehicle or machine equipment	208	16.0	15	16.9	193	16.0	
Other	75	5.8	3	3.4	72	6.0	
Mode of transportation							<0.0001
EMS use	888	68.4	78	87.6	810	67.0	
In-hospital mortality							<0.0001
Death	39	3.0	9	10.1	30	2.5	
Expected days away from work							<0.0001
Median (IQR)	21 (7–42)		33 (21–56)		21 (6–42)		
Convalescence by insurance, week							0.001
Median (IQR)	6.0 (2–8)		7.5 (6–8)		6.0 (2–8)		

IQR: interquartile range.

**Table 3:** Logistic regression analysis on study outcomes by safety helmet and height of fall injury.

Characteristics	Total	Outcomes		Unadjusted		Adjusted ^†^	
	*N*	*n*	%	OR	95% CI	OR	95% CI
**Primary Outcome: Intracranial Injury**							
Helmet							
Safety helmet	584	27	4.6	0.51	(0.32–0.81)	0.42	(0.24–0.73)
No safety helmet	714	62	8.7	1.00		1.00	
Height of fall, meter							
0–2	370	18	4.9	1.00		1.00	
2–4	595	44	7.4	1.56	(0.89–2.75)	1.65	(0.91–2.98)
4–6	192	15	7.8	1.66	(0.82–3.37)	1.73	(0.82–3.67)
Over 6	141	12	8.5	1.82	(0.85–3.88)	2.24	(1.00–5.04)
**Secondary Outcome: In-Hospital Mortality**							
Helmet							
Safety helmet	584	18	3.1	1.05	(0.55–1.99)	0.83	(0.34–2.03)
No safety helmet	714	21	2.9	1.00		1.00	
Height of fall, meter							
0–2	370	3	0.8	1.00		1.00	
2–4	595	10	1.7	2.09	(0.57–7.65)	3.11	(0.80–12.09)
4–6	192	8	4.2	5.32	(1.40–20.29)	10.27	(2.42–43.54)
Over 6	141	18	12.8	17.9	(5.19–61.81)	39.54	(9.84–158.88)

**^†^** Adjusted for age, gender, education level, annual income, occupation, safety education enrollment status, type of employment, working type, time of injury, and place of injury. OR: odds ratio; 95% CI: 95% confidence interval.

**Table 4:** Effects of safety helmet in an interaction model with the height of fall injury.

Characteristics	Total	Outcomes		Adjusted ^†^	
	*N*	*n*	%	OR	95% CI
**Primary Outcome: Intracranial Injury**					
Height of fall, 0–2 m					
Safety helmet	116	2	1.7	0.21	(0.05–0.96)
No safety helmet	254	16	6.3	1.00	
Height of fall, 2–4 m					
Safety helmet	275	12	4.4	0.37	(0.18–0.77)
No safety helmet	320	32	10.0	1.00	
Height of fall, 4–6 m					
Safety helmet	100	6	6.0	0.64	(0.21–1.98)
No safety helmet	92	9	9.8	1.00	
Height of fall, over 6 m					
Safety helmet	93	7	7.5	0.74	(0.21–2.67)
No safety helmet	48	5	10.4	1.00	
**Secondary Outcome: In-hospital Mortality**					
Height of fall, 0–2 m					
Safety helmet	116	0	0.0	-	-
No safety helmet	254	3	1.2	1.00	
Height of fall, 2–4 m					
Safety helmet	275	4	1.5	0.99	(0.25–3.91)
No safety helmet	320	6	1.9	1.00	
Height of fall, 4–6 m					
Safety helmet	100	4	4.0	1.20	(0.25–5.69)
No safety helmet	92	4	4.3	1.00	
Height of fall, over 6 m					
Safety helmet	93	10	10.8	0.70	(0.19–2.56)
No safety helmet	48	8	16.7	1.00	

^†^ Adjusted for age, gender, education level, annual income, occupation, safety education enrollment status, type of employment, working type, time of injury, place of injury, and interaction term (safety helmet × height of fall). OR: odds ratio; 95% CI: 95% confidence interval.
